# Supplementary material for: Genome-Wide Biases in the Rate and Molecular Spectrum of Spontaneous Mutations in Vibrio cholerae and Vibrio fischeri
Source: Mol Biol Evol. 2016 Oct 15;34(1):93–109. doi: 10.1093/molbev/msw224 (PMC5854121; doi:10.1093/molbev/msw224)
Supplement: Supplementary Data [file msw224_suppl.zip › VibrioMutation_SupplementalMerged.docx]

**Genome-wide biases in the rate and molecular spectrum of spontaneous mutations in *Vibrio cholerae* and *Vibrio fischeri***

Marcus M. Dillon, Way Sung, Michael Lynch, Robert Sebra, and Vaughn S. Cooper

Supplemental Information

**Supplemental Text**

Selection in MA Experiments

The properties of our MA experiments allow us to assume that few mutations are subject to the biases of natural selection. The threshold selective coefficient (*s*) below which genetic drift will overpower natural selection is determined by $N_{E} \times s =1$ (Lynch 2007), where $N_{E}$ is the effective population size. By estimating $N_{E}$ for each MA experiment using the harmonic mean of the population size (*N*) (Hall et al. 2008), we infer that only mutations conferring an adaptive or deleterious effect (*s*) greater than 0.083, 0.067, 0.106, and 0.069 for *Vf*-wt, *Vc*-wt, *Vf*-mut, and *Vc*-mut, respectively, will be subject to the biases of natural selection. This is expected to be a very small fraction of mutations (Kimura 1983; Hall et al. 2008). Furthermore, if we exclude indels that were identified at the same site or SSR, only four genes were hit more than once and no genes were hit more than twice across all wild-type lineages, suggesting that strong positive selection acting on common traits did not influence these experiments.

Other metrics that have been used to test that the efficiency of purifying selection is minimized in MA experiments include the ratio of coding to non-coding mutations and the ratio of synonymous to nonsynonymous bpsms. However, both of these tests are problematic as preferential mismatch repair in coding regions (Lee et al. 2012), context-dependent mutation biases (Sung et al. 2015), and a non-uniform distribution of mutation rates and spectra across the genome (Foster et al. 2013; Dillon et al. 2015; Dettman et al. 2016) can generate artificial signatures of natural selection. These issues are evident in our MA experiments, where chi-square tests comparing our observed mutations with the expected ratios of coding to non-coding DNA and synonymous to nonsynonymous sites in each genome were at times inconsistent. In the *Vf*-wt lines, we observe an excess of non-coding indels and bpsms (Bpsm: χ^2^ = 4.006, d.f. = 1, p = 0.045, Indels: χ^2^ = 61.434, d.f. = 1, p < 0.0001), while the ratio of nonsynonymous to synonymous bpsms did not differ significantly from the null expectation (χ^2^ = 0.907, d.f. = 1, p = 0.341). In the *Vc*-wt lines, non-coding bpsms are again in excess (χ^2^ = 8.739, d.f. = 1, p = 0.003), while the ratio of coding to non-coding indels (χ^2^ = 1.479, d.f. = 1, p = 0.224) and nonsynonymous to synonymous bpsms (χ^2^ = 1.469, d.f. = 1, p = 0.226) do not differ from the null expectation. The excess of non-coding indels and bpsms could imply that selection played a small role in eradicating coding mutations or that mismatch repair is more active in coding regions, while all other lines of evidence support that minimal selection was operating in our wt MA experiments.

In the *Vf*-mut lines, we observe an excess of coding bpsms (χ^2^ = 39.083, d.f. = 1, p < 0.0001) and non-coding indels (χ^2^ = 206.820, d.f. = 1, p < 0.0001), but the ratio of nonsynonymous to synonymous bpsms does not differ from the null expectation (χ^2^ = 2.534, d.f. = 1, p = 0.111). In the *Vc*-mut lines, we observe an excess of non-coding indels (χ^2^ = 123.710, d.f. = 1, p < 0.0001) and synonymous bpsms (χ^2^ = 5.595, d.f. = 1, p = 0.018), while the ratio of coding to non-coding substitutions does not differ from the null expectation (χ^2^ = 2.354, d.f. = 1, p = 0.125). The excess of coding bpsms and non-coding indels observed in the *Vf*-mut MA experiment suggest contradictory forms of selection, since an excess of coding bpsms is a signature of positive selection, and an excess of non-coding indels is a signature of purifying selection. Both the excess of non-coding indels and synonymous substitutions observed in the *Vc*-mut MA experiment suggest the operation of purifying selection. Altogether, these observations suggest that selection may have played a small role in eliminating some mutations during the mutator MA experiments, however, we should not rule out the possibility that these signatures of selection were generated by non-adaptive mutational biases (Foster et al. 2013; Dillon et al. 2015; Sung et al. 2015; Dettman et al. 2016).

**Supplemental Tables**

**Table S1.** Conditional base-substitution mutation (bpsm) rates for wild-type *Vibrio fischeri* and *Vibrio cholerae* in different replication timing regions. Early chr1 regions are regions on chr1 replicated prior to the initiation of chr2 replication, late chr1 regions are regions on chr1 replicated concurrently with chr2, and chr2 regions are the bpsm rates on chr2 itself.

| Species | Bps Type | Early chr1 | | Late chr1 | | Chr2 | |
| --- | --- | --- | --- | --- | --- | --- | --- |
|  |  | Avg | SEM | Avg | SEM | Avg | SEM |
| *V. fischeri* | A:T > G:C | 4.25×10^-11^ | 1.37×10^-11^ | 3.57×10^-11^ | 1.28×10^-11^ | 5.43×10^-11^ | 1.47×10^-11^ |
|  | G:C > A:T | 1.69×10^-10^ | 3.24×10^-11^ | 1.43×10^-10^ | 3.74×10^-11^ | 2.24×10^-10^ | 4.43×10^-11^ |
|  | A:T > T:A | 4.25×10^-12^ | 4.30×10^-12^ | 9.78×10^-12^ | 6.91×10^-12^ | 4.80×10^-12^ | 4.85×10^-12^ |
|  | G:C > T:A | 1.10×10^-10^ | 2.73×10^-11^ | 1.98×10^-10^ | 4.12×10^-11^ | 2.78×10^-10^ | 4.86×10^-11^ |
|  | A:T > C:G | 3.83×10^-11^ | 1.18×10^-11^ | 3.91×10^-11^ | 1.47×10^-11^ | 3.84×10^-11^ | 1.44×10^-11^ |
|  | G:C > C:G | 4.54×10^-11^ | 1.87×10^-11^ | 1.58×10^-11^ | 1.12×10^-11^ | 8.17×10^-12^ | 8.26×10^-12^ |
| *V. cholerae* | A:T > G:C | 3.89×10^-11^ | 1.10×10^-11^ | 2.74×10^-11^ | 1.18×10^-11^ | 1.08×10^-11^ | 7.67×10^-12^ |
|  | G:C > A:T | 1.12×10^-10^ | 1.85×10^-11^ | 7.92×10^-11^ | 2.11×10^-11^ | 1.23×10^-10^ | 2.50×10^-11^ |
|  | A:T > T:A | 9.72×10^-12^ | 5.55×10^-12^ | 5.47×10^-12^ | 5.52×10^-12^ | 1.08×10^-11^ | 7.66×10^-12^ |
|  | G:C > T:A | 4.18×10^-11^ | 1.18×10^-11^ | 3.05×10^-11^ | 1.59×10^-11^ | 4.93×10^-11^ | 1.63×10^-11^ |
|  | A:T > C:G | 1.94×10^-11^ | 8.91×10^-12^ | 2.74×10^-11^ | 1.18×10^-11^ | 3.25×10^-11^ | 1.68×10^-11^ |
|  | G:C > C:G | 6.97×10^-12^ | 4.93×10^-12^ | 6.10×10^-12^ | 6.16×10^-12^ | 1.85×10^-11^ | 1.05×10^-11^ |

**Table S2.** Relative frequencies of insertion-deletion mutations (indels) observed in the wild-type and mutator mutation accumulation experiments with *Vibrio fischeri* and *Vibrio cholerae*. Chi-square tests were conducted to test whether indels in each size category were significantly over-represented in the mutator lineages after correcting for differences in total number of sites analyzed across all lineages and the number of generations in the wild-type and mutator experiments.

| Species | Indel | Indels Observed | | Expected Frequencies | | χ^2^ | df | p |
| --- | --- | --- | --- | --- | --- | --- | --- | --- |
|  | Length | Wt | Mut | Wt | Mut |  |  |  |
| *V. fischeri* | 1 | 12 | 352 | 0.942 | 0.058 | 5460.000 | 1 | <0.0001 |
|  | 2 | 0 | 11 | 0.942 | 0.058 | 177.200 | 1 | <0.0001 |
|  | 3 | 0 | 5 | 0.942 | 0.058 | 80.542 | 1 | <0.0001 |
|  | 4 | 1 | 0 | 0.942 | 0.058 | 0.062 | 1 | 0.803 |
|  | 5 | 2 | 0 | 0.942 | 0.058 | 0.124 | 1 | 0.725 |
|  | 6 | 4 | 2 | 0.942 | 0.058 | 8.238 | 1 | 0.004 |
|  | 7 | 19 | 4 | 0.942 | 0.058 | 5.572 | 1 | 0.018 |
|  | 8 | 3 | 0 | 0.942 | 0.058 | 0.186 | 1 | 0.666 |
|  | 9 | 0 | 0 | 0.942 | 0.058 | - | - | - |
|  | 10 | 0 | 2 | 0.942 | 0.058 | 32.217 | 1 | <0.0001 |
| *V. cholerae* | 1 | 8 | 233 | 0.920 | 0.080 | 2567.000 | 1 | <0.0001 |
|  | 2 | 0 | 30 | 0.920 | 0.080 | 343.900 | 1 | <0.0001 |
|  | 3 | 2 | 7 | 0.920 | 0.080 | 59.332 | 1 | <0.0001 |
|  | 4 | 2 | 0 | 0.920 | 0.080 | 0.174 | 1 | 0.676 |
|  | 5 | 0 | 0 | 0.920 | 0.080 | - | - | - |
|  | 6 | 0 | 1 | 0.920 | 0.080 | 11.462 | 1 | 0.001 |
|  | 7 | 0 | 0 | 0.920 | 0.080 | - | - | - |
|  | 8 | 0 | 0 | 0.920 | 0.080 | - | - | - |
|  | 9 | 1 | 1 | 0.920 | 0.080 | 4.775 | 1 | 0.029 |
|  | 10 | 2 | 0 | 0.920 | 0.080 | 0.174 | 1 | 0.676 |

**Supplemental Figures**

**Figure S1.** Estimates of the average number of generations per day experienced by the *Vibrio fischeri* wild-type, *Vibrio cholerae* wild-type, *Vibrio fischeri* *ΔmutS*, and *Vibrio cholerae* *ΔmutS* mutation accumulation lineages. Each measurement was taken using the average of ten representative lineages per MA experiment and measurement error is such that error bars representing 95% confidence intervals are not visible outside of the markers.

**Figure S2.** Relationship between base-substitution mutation (bpsm) rate and insertion-deletion (indel) rate per effective genome size per generation with effective population size (N_E_). Four multicellular eukaryotes are shown in red, three unicellular eukaryotes are shown in black, and eight prokaryotes are shown in blue (Sung et al., 2016). *Vibrio fischeri* and *Vibrio cholerae* wild-type bpsm and indel rates rates estimated in this study are highlighted in green. The log-linear regressions are highly significant for both bpsm rate (r^2^ = 0.86, p < 0.0001, df = 14) and indel rate (r^2^ = 0.94, p < 0.0001, df = 14).
